# Pricing and procurement strategies in the relief supply chain via bidirectional option contract

**DOI:** 10.1371/journal.pone.0341427

**Published:** 2026-04-01

**Authors:** Mahsa Maleki Rastaghi, Farnaz Barzinpour, Jafar Heydari, Mohammad Reza Gholamian

**Affiliations:** 1 School of Industrial Engineering, Iran University of Science and Technology, Tehran, Iran; 2 Department of Quantitative Studies, School of Management and Business, University Canada West, Vancouver, British Columbia, Canada; SRMIST: SRM Institute of Science and Technology (Deemed to be University), INDIA

## Abstract

Humanitarian supply chain management is a critical and challenging issue for all members of the supply chain due to the high uncertainty of the demand caused by a disaster and the direct connection of this type of supply chain with the well-being of the affected individuals. One strategy to increase supply flexibility and reduce the risk of inventory shortages or surpluses in humanitarian supply chains is to use option contracts. In this study, we investigate the optimization of decisions in a two-echelon humanitarian supply chain consisting of a humanitarian organization and a supplier through a bidirectional option contract, considering the perspectives of both supply chain members. We rely on realistic assumptions regarding the relief supply chains, such as the occurrence probability of disasters and differing salvage values of the supplier and the humanitarian organization. In addition, the optimal decisions of the humanitarian organization and supplier are extracted based on the Stackelberg game. Then, we demonstrate that when the humanitarian organization’s salvage value is equal to the supplier’s, the bidirectional option contract becomes a call option contract. Under the same condition, optimal procurement and pricing strategies of the humanitarian organization and the supplier are extracted. Finally, we show through numerical examples that the performance of the proposed model more closely resembles that of the centralized model than the wholesale price model in improving the supply chain members’ objectives. The results indicate that using a bidirectional option contract leads to improvements in supply chain members’ performance and can help achieve channel coordination.

## 1. Introduction

**Fig 1:**
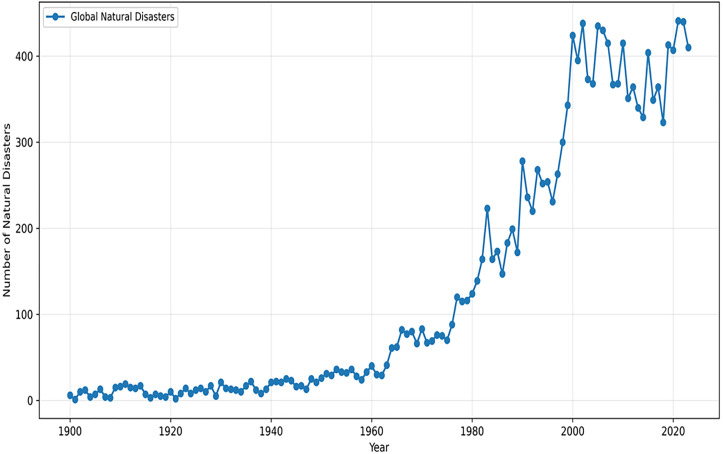
Number of recorded global natural disaster events, 1900 to 2023 [3].

A disaster is one of the most significant events that may occur in any society at any given moment. Disasters typically occur suddenly and without warning, are accompanied by extensive human and economic losses, and impose complex difficulties on society and organizations, which should be mitigated through swift and urgent measures [1]. According to the data provided by the International Disaster Database (EM-DAT [2]), about two-thirds of all recorded disasters worldwide are related to natural hazards. Fig 1 illustrates the global number of natural disasters between 1900 and 2023. As can be seen, the number of natural disasters has markedly increased, especially in the last decade, likely due to challenges such as population growth and climate change.

The response phase plays a key role in rescuing victims and preventing further destruction of buildings and infrastructure in the aftermath of a major disaster. Developing and evaluating new approaches that shorten the response time in delivering relief items can significantly contribute to protecting lives and reducing damage to property during disasters such as earthquakes [4]. Given the importance of this phase and the short time window within which the post-disaster response should be initialized, a great deal of the literature on emergency management has been focused on relief supply chains [5].

Humanitarian supply chains (HSCs) are a specialized type of supply chain responsible for providing the basic needs of those affected by disasters as quickly and appropriately as possible. HSCs are often characterized by uncertain demand patterns, multiple parallel supply chains, inadequate infrastructure in affected areas, and other complexities [6]. Procurement of the relief items is a critical part of the humanitarian logistics and if done effectively, it can help save the lives of the many injured people [7].

A humanitarian supply chain (HSC) is activated when a disaster occurs, and its operational characteristics vary depending on the type of crisis and the various actors involved. In an HSC, HO’s (humanitarian organization’s) usually pre-position the relief items during the pre-disaster phase and distribute them to affected areas during the post-disaster phase, especially within the first 72 hours following a disaster event [8]. In this case, HO’s may face inventory or shortage risks. As regards the risk of shortages, let us consider the instance where, around 48 hours following the Wenchuan earthquake, there was a grave shortage of roughly one million tents within the impacted region, resulting in approximately 300 individuals having to share a single tent in certain areas. As for the risks associated with inventory, take the example of the situation involving the Federal Emergency Management Agency (FEMA). Following the criticism this organization received due to its failure to provide adequate supplies in response to Hurricane Katrina in 2005, FEMA this time accumulated excessive relief provisions in 2006. However, since there were no major hurricanes that year, FEMA ended up losing 279 truckloads of perishable foods valued at approximately $43 million [9]. Due to the high uncertainty in emergency situations, buyers in HSCs, such as non-profit organizations, should use special tools, such as contracts, to take the uncertainty under control and mitigate its impact on the supply chain. Leveraging contracts strengthens HO’s flexibility and resilience, extending their advantages to disaster response and post-disaster preparedness [10].

Option contracts, including call options, put options, and bidirectional options, have been used for a long time as a financial derivatives to prevent risks and reduce uncertainties in financial affairs [11]. Numerous studies on supply chains in various industries, including apparel [12], electronics [13], service leasing [14], perishable food products [15–18], and crisis management [19,20] have demonstrated that option contracts provide sufficient flexibility to retailers in terms of procurement and ordering to satisfy unexpected demand. This is because option contracts enable retailers to decide whether to purchase more items or return their remaining inventory to the supplier after actual demand information becomes available. Moreover, they attract suppliers because they earn income at the start of the contract without having offered any goods yet [10–12]. Additionally, on the basis of this early commitment, suppliers can ensure better planning in terms of their capacity and materials. Therefore, option contracts create profit for both chain members [21].

Given the flexibility of bidirectional option contracts, which offer the simultaneous benefits of both the call and put options, or in simpler terms, the possibility to buy or sell based on the HO’s decision, the authors got interested in using this type of contract in an HSC to reduce the inventory and shortage risks common in such networks. In the real world, when a bidirectional option contract is used in an HSC, the goal is to protect the interests and address the concerns of both the HO and the supplier. To be specific, the HO needs to make decisions regarding the supply of relief items to the affected areas, while the supplier’s main concern is to set prices that not only attract the HO but also increase its own profit. Suppliers can have a considerable influence on the adequate distribution of relief provisions under option contracts [22]. However, studies that have been found in the literature in the field of using bidirectional option contracts in the HSCs have focused on optimal decision-making by the HOs and have not paid attention to optimizing suppliers’ decisions [15,16] and the prices offered by the supplier in the contract have been considered as parameters.

When it comes to planning relief supply chains with bidirectional option contracts, there are several assumptions that may have direct or indirect effects on the decision-making of all chain members. For instance, the occurrence probability of a disaster for an HO is seldom 100%. Rather, it may even be zero in certain areas, and the HO must base its plans and decisions on the probability of disasters. Additionally, in a supply chain featuring bidirectional option contracts, the amount of supply reserved for call options by the HO may not always be equal to the amount reserved for put options. These values often differ and can be defined and optimized as independent decision variables. Moreover, the exercise prices and reservation prices of the call and put options are not the same, and the salvage values associated with the HO and the supplier are not equal, either. However, prior studies on bidirectional option contracts in the context of relief supply chains have often simplified these assumptions.

Through our collaboration with the Iranian Red Crescent Society, it has been observed that many non-profit organizations managing natural disasters, such as floods and earthquakes, employ bidirectional option contracts to effectively mitigate inventory and shortage risks. For example, mineral water—a critical relief item—is specifically procured under such contracts in disaster management operations. Furthermore, in planning relief supply chains with bidirectional option contracts, the probability of disaster occurrence—which varies across different provinces and types of disasters—is explicitly incorporated. Additional real-world considerations, including differing salvage values for the HO and supplier, as well as varied reservation prices for call and put options, are also inherent features of these supply chains.

In light of these observed facts in Iran, along with the complexities and gaps in the existing literature, this study aims to address the following research questions:

1- How can bidirectional option contracts be used to reduce inventory and shortage risks in humanitarian supply chains?
2- How can the problem be modeled considering real-world assumptions, such as different reserved quantities for call and put options, in humanitarian supply chains using bidirectional option contracts?
3- What are the optimal procurement and pricing decisions for both the HO and the supplier in the proposed model?
4- Do bidirectional option contracts improve the objective values of supply chain members and enhance coordination between them?

To answer these questions, this study proposes a two-echelon HSC consisting of a supplier and an HO. We employ bidirectional option contracts based on real-world assumptions. We have addressed the optimization of this problem from the perspective of both HSC members. By employing Stackelberg games, we not only optimize the decisions of the HO but also extract the contract prices proposed by the supplier to the HO. Numerous researchers (e.g., [8,18,22,23] have evaluated the performance of their proposed option contract models by comparing them with similar problems involving wholesale price contracts in the supply chain. In light of this trend in the literature, we developed a centralized and a wholesale price contract model and compared its performance with our proposed bidirectional option contract. The main contributions of our study are:

- The development of an HSC model incorporating a bidirectional option contract that conforms to the assumptions of real-world scenarios, including the occurrence probability of disasters, different salvage value for the supplier and the HO, different reserving call and put options, and the varying prices of exercising the call and put options.
- The examination of HSC management from the perspective of both chain members simultaneously and use the Stackelberg game to solve the proposed problems.
- The derivation of the HO’s optimal strategies for relief item procurement and the optimal pricing of the supplier’s proposed contract with a bidirectional option contract.
- The discussion of mathematically and numerically the effect of parameters such as salvage value, occurrence probability of disasters, exercise price of the HO’s and the supplier’s decision-making and performance, and report interesting results and managerial implications.

The remainder of this study is organized as follows: Section 2 reviews the literature related to the topic discussed in this study. Section 3 defines the problem and states the assumptions and notations. Section 4 presents the three models addressed in this study, including the proposed bidirectional option contract model, the benchmark model, and the centralized model, along with the optimal decisions of each model. Section 5 presents the sensitivity analysis performed on parameters and evaluates the performance of the proposed model compared to the benchmark and centralized models. Finally, Sections 6 and 7 present the managerial implications derived from our findings, a brief conclusion, and suggestions for future research.

## 2. Literature review

In the context of using the option contract in supply chains, it should be noted that, firstly, this type of contract has been used by researchers in the commercial supply chain and many works have been published in the field of call option contracts [19–28], put option contracts [29–33] and bidirectional option contracts, [34–39]. Since the focus of the present study is on the use of option contracts in a relief supply chain, we first present the details of the reviewed articles on the use of option contracts in the HSC in Section 2.1, and then outline the existing research gaps in Section 2.2.

### 2.1. Option contract in the HSC

In the context of HSCs, option contracts have emerged as valuable tools to manage procurement uncertainties, inventory risks, and coordination issues between HOs and suppliers. The following subsections summarize the use of different types of option contracts (call, put, and bidirectional) in HSCs, highlighting key studies in each area.

- **Call Option Contract in the HSC**

In the context of HSCs that make use of call option contracts, Liang et al. [11] proposed a binomial pricing model for a two-echelon relief supply chain, and extracted the feasible price range of the option contract. Wang et al. [40] examined a pre-disaster procurement model for relief items using a call option contract and demonstrated the superiority of their proposed model over two benchmark models.

Rabbani et al. [5] presented a binomial tree-based pricing mechanism to determine the range of option and exercise prices. Shamsi G. et al. [22] developed a model based on the SIR (Susceptible, Infectious, and Removed) epidemiological model in the vaccine supply chain to minimize social costs and the buyer’s procurement costs, as well as maximize suppliers’ profit. Liu et al. [23] developed a relief item procurement model using a call option contract and calculated the optimal order quantity for the government and the inventory level for each supplier using a decision-making game based on the Stackelberg game.

Aghajani et al. [21] addressed the procurement problem in an HSC under supplier and warehouse disruptions. The researchers developed a bi-objective model by integrating the call option contracts with the supplier selection problem (maximum coverage) and pre-disaster prepositioning. Lastly, Akbarpour et al. [41] designed an integrated relief network for the prepositioning of essential pharmaceutical products prior to the disaster and distribution of essential and non-essential medicines after the occurrence of the disaster using a call option contract.

John et al. [42] studied how using a call option contract in the procurement of relief items can reduce inventory risk for buyers and over-production risk for suppliers. By developing mathematical models and extracting optimal decisions for members, they determined the price range for the contract that would facilitate coordination among members of the supply chain. Liu et al. [43] divided relief items into two categories, consumable and non-consumable. Then, using a call option contract, they analyzed characteristics of each item type, and by developing two separate models identified the optimal inventory levels for the buyer and production quantities for the supplier.

Liu et al. [44] investigated the procurement and supply of relief items in an HSC involving a government and a loss-averse supplier with anchoring. In addition to determining the initial government order and the level of emergency production by the supplier, the conditions under which the chain members achieve optimal coordination were also extracted. Fan et al. [45] addressed the collaboration between a relief organization and a private sector vendor in an HSC to solve procurement and inventory issues. Taking into account the HO’s objectives and the necessity of inventory management, the authors proposed nine models incorporating a call option contract and compared them using numerical examples.

Yu et al. [46] developed a model of production capacity reserves considering a call option contract, in which production capacity can be rapidly converted into tangible products to meet emergency demands. The study examines a loss-averse supplier and addresses the determination of both the reserve capacity level and the emergency option order quantity, highlighting the importance of capacity management under demand uncertainty. Recently, Peng et al. [47] investigated three contractual mechanisms to enhance material reserves during emergencies: the call option contract (OP), the call option contract with revenue-sharing (RS), and the call option contract with price-discount (PD). They evaluated these models across different scenarios and highlighted the conditions under which each contract emerges as the most suitable strategy

- **Put Option Contract in the HSC**

As far as our review of the literature on option contracts shows, Hu et al. [9] have been the only researchers to have used a put option contract for the procurement of relief items to reduce the buyer’s inventory risks without negatively affecting suppliers’ profit. To demonstrate the effectiveness of the put option contract, the authors compared this arrangement under identical conditions with a wholesale price contract and a buyback contract.

• **Bidirectional Option Contract in the HSC**

Regarding the use of bidirectional option contracts in relief supply chains, Patra and Jha [19] worked on a two-echelon supply chain consisting of a supplier and an HO, optimizing the decisions from the HO’s perspective and extracting the closed-form of the HO’s optimal decisions while taking into account both uniform and exponential distributions. In a similar study, Meng et al. [20] optimized the HO’s decisions using a bidirectional option contract and the possibility of purchasing from the spot market in case of facing additional demand in a relief supply chain.

### 2.2. Research gap

In order to better clarify the most prominent research gaps, the existing studies on HSCs that have used option contracts are summarized in Table 1, which reveals several gaps within the literature on HSCs with option contracts:

**Table 1:** Comparative analysis of the current study and reviewed literature on humanitarian supply chains incorporating different option contracts (call, put, bidirectional).

Reference	Year	Type of option contract			Assumptions				Perspective		Decision variables			
		Call	Put	Bidirectional	Probability of disaster occurrence	Salvage value for parties	Exercise prices	Reserved options	Buyer	Supplier	Initial order	Option order	Call option price	Put option price
Liang et al.	2012	✓				NA	NA	NA	✓	✓			✓	NA
Wang et al.	2015	✓				NA	NA	NA	✓		✓	✓		NA
Rabbani et al.	2015	✓				NA	NA	NA	✓	✓			✓	NA
Shamsi et al.	2018	✓			✓	S	NA	NA	✓	✓	✓		✓	NA
Liu et al.	2019	✓			✓	S	NA	NA	✓	✓	✓			NA
Hu et al.	2019		✓			S	NA	NA	✓		✓	✓	NA	
Aghajani et al.	2020	✓				NA	NA	NA	✓		✓	✓		NA
Akbarpour et al.	2020	✓				NA	NA	NA	✓		✓			NA
John et al.	2022	✓				S	NA	NA	✓	✓	✓	✓		NA
Liu et al.	2022	✓			✓	S	NA	NA	✓	✓	✓	✓		NA
Patra & Jha	2022			✓		S	D	S	✓		✓	✓		
Meng et al.	2023			✓		S	S	S	✓		✓	✓		
Liu et al.	2023	✓			✓	S	NA	NA	✓	✓	✓	✓		NA
Fan et al.	2024	✓				S	NA	NA	✓	✓	✓	✓		
Yu et al.	2025	✓			✓	S	NA	NA	✓	✓		✓		
Peng at al.	2025	✓			✓	NA	NA	NA	✓			✓		
This research	2025			✓	✓	D	D	D	✓	✓	✓	✓	✓	✓

**NA**: Not applicable to this research; **S**: Considering the same value for this assumption; **D**: Considering a different value for this assumption.

- To the best of our knowledge, no existing study has addressed the optimization of contract pricing for suppliers using a bidirectional option contract. Traditionally, researchers tend to treat the reservation prices of call and put options as parameters (e.g., [18,19]).
- In the real world, the likelihood of a disaster such as an earthquake varies across different regions, and this significantly influences the optimal solutions of the HO and suppliers [48]. Surprisingly, none of the reviewed articles have addressed this crucial factor when discussing the utilization of bidirectional option contracts in relief supply chains.
- Another real-world assumption is that the salvage value of an item is invariably higher for the supplier than for the HO [21,22,42,43]. This is because the supplier can sell any leftover items to a second customer at a lower price; while the HO, lacking a market, must salvage redundant items at a lower price than the supplier. However, neglecting this key difference, researchers in the field of bidirectional option contracts in relief supply chains have assumed equal salvage values to simplify the problem.
- In the real world, when a bidirectional option contract is adopted, the reservation and exercise prices of the call option are not always equal to those of the put option. In commercial supply chains, such as the one discussed in Wan and Chen [49], different reservation and exercise prices for call and put options are considered. However, to the best of our knowledge, only Patra and Jha [19] have set different exercise prices for the call and put options, and the same reservation prices, under disaster conditions.
- Moreover, the reserved option amount for the call can differ from that of the put, as mentioned in Wan and Chen [49]. However, the articles pertaining to bidirectional option contracts in HSCs tend to simplify these two orders by considering and optimizing a single decision variable.

Therefore, based on the literature reviewed for the purposes of this study and above research gaps, our goal is to employ a bidirectional option contract in an HSC, examine the problem from the perspective of both chain members, and optimize their decisions by taking into account realistic assumptions, such as the uncertain nature of the occurrence of disasters, different salvage values for the HO and supplier, different option reservation prices, different exercise prices, and different option orders for call and put options during the contract period. The subject addressed in this research will be discussed in detail in the following sections.

## 3. Problem definition

This study considers a two-echelon, single-period HSC consisting of an HO and a supplier. The HO procures the necessary items from the supplier in the pre-disaster phase and distributes them in affected areas in the event of a disaster. Due to the considerable time required to manufacture the required products and the short time window (less than 72 hours; known as the ‘golden time’) for responding to the surging demand after the occurrence of a disaster, the HO can place orders with the supplier only once before the disaster occurs. At the end of the planning horizon, all remaining items in the inventory should be salvaged by the HO at a lower rate than the original procurement price of the items. All the symbols used in this study are summarized in Table 2.

**Table 2:** Notations used in this study.

Notations	Description
**Parameters**	
x	Random variable representing demand after the disaster with probability density function f(x) and cumulative density function F(x)
w	The unit wholesale price ($ per unit)
c	The unit manufacturing cost of the supplier ($ per unit)
π	The probability of occurrence of the disaster, with values in the interval [0, 1]
g	The unit shortage cost ($ per unit)
$v_{b}$	The unit salvage value for the HO ($ per unit)
$v_{s}$	The unit salvage value for the supplier ($ per unit)
$e_{c}$	The unit exercise price for call option ($ per unit)
$e_{p}$	The unit exercise price for put option ($ per unit)
*b*	The upper‐bound estimate of the population affected by the disaster, assuming x follows a uniform distribution on [0,b]
λ	The weighting parameter in [0,1], defining the convex combination of two parameters.
**Decision variables**	
$Q_{w}$	The wholesale order quantity in the benchmark model
$Q_{cs}$	The order quantity in the centralized system
$Q_{BO}$	The initial order quantity for prepositioning under bidirectional option contract
$q_{c}$	The reserved call option quantity under bidirectional option contract
$q_{p}$	The reserved put option quantity under bidirectional option contract
$o_{c}$	The call option price per unit ($)
$o_{p}$	The put option price per unit ($)

The model is built upon a set of assumptions. Assumptions 1–6 and 13 are grounded in prior HSC studies, with additional references incorporated to reinforce their validity. Assumptions 7–12 are newly proposed in this study to better capture realistic operational and economic conditions and to highlight the theoretical contributions of our work. The assumptions are presented as follows:

1. The Stackelberg game, a strategic game in which the leader moves first and the follower responds optimally, is employed to determine the optimal decisions of the two chain members in the studied HSC, as explored in previous studies such as [14,50,51,52]. This game is solved using backward induction: first, the follower’s optimal response is derived given the leader’s decision, and then the leader optimizes its decision based on the follower’s response. Accordingly, in the proposed HSC model reflecting real-world conditions, the supplier acts as the leader, making the first move before the disaster occurs and proposing contract prices, while the HO acts as the follower, responding optimally based on the information provided by the supplier.
2. According to previous works, especially [15,16,50,51], the contract specifies a shorter deadline than the expiration dates of the relief items. Consequently, any remaining items in the inventory will be salvaged at a reduced price upon the contract’s expiration.
3. Due to the occurrence of the hypothetical disaster, the demand in affected areas is considered uncertain and stochastic. For extracting optimal decisions, we utilize a uniform probability distribution function when appropriate, a practice supported by previous studies (such as [7,23,43]) in the field of relief supply chains.
4. Since the typical shelf life of humanitarian aid goods, such as water or foodstuffs, in warehouses is less than two years, the time value of money is not factored into the mathematical modeling.
5. $o_{c}+e_{c}\leq g$: given the nature of the HSC, the potential inability of the HO to meet certain demands can pose significant life and financial risks. As a result, the HO’s primary concern is to make order decisions that ensure the fulfillment of all demands. In this context, it has been observed in previous researches, including [15,16,51] that the cost of reserving and exercising the call option consistently remains lower than the per-unit shortage cost, which represents the cost incurred for each instance of failure to respond to demand.
6. $o_{p}+v_{b}\leq e_{p}$: This condition ensures that reserving and exercising the put option, if necessary, is more profitable for the HO than salvaging the items. This has been confirmed by [8,18,19] as well.
7. Since the probability of disaster occurrence varies across different regions and this probability directly influences decision-making, unlike previous studies, the problem is modeled based on the probability of disaster occurrence.
8. The salvage value of relief items for the HO is lower than that for the supplier, as the supplier is better recognized in the market and can salvage the items at a higher value. Therefore, unlike previous studies, the salvage values for the supplier and the HO are considered different in the proposed HSC.
9. In real-world settings, the quantities of reserved call and put options at the time of bidirectional option contract formation are not necessarily equal. Therefore, the model considers two distinct variables for the reservation quantities of call and put options.
10. The reservation and exercise prices of call options are generally not equal to those of put options in real-world scenarios; therefore, these prices are modeled separately in this study.
11. $e_{c}=\lambda w+(1-\lambda )g$: Exercising each unit of the call option in the post-disaster phase imposes higher costs relative to purchasing relief items at wholesale prices prior to the disaster. Moreover, according to assumption 5, a linear combination of the maximum value and minimum value of the exercise price of the call option, such as a buyback contract [53], is considered as the price of exercising the call option.
12. $e_{p}=\lambda v_{b}+w(1-\lambda )$: From the HO’s perspective, it is imperative to prevent the unnecessary purchase of emergency items and their subsequent return. This is manifested in the condition that $e_{p}\leq w$. Similarly, from the supplier’s perspective, it is essential to maintain $e_{p}\leq w$ to ensure that the cost incurred by the supplier for each unit of returned items resulting from the exercise of the put option by the retailer is lower than the wholesale price. This guarantees that this particular aspect of the bidirectional option contract is attractive to the supplier. Assumption 7 states that ($v_{b}<e_{p})$. Therefore, taking into account the minimum and maximum values of $e_{p}$, a linear combination of these values is considered as the exercise price of the put option.
13. $v_{b}<v_{s}<c<w<g$: This constraint indicates the actual relations between other costs in the studied supply chain. Since the supplier must earn a profit for selling each item, Furthermore, it ensures that the salvage value of each item is invariably higher for the supplier than the HO. Finally, it states that the HO’s main objective is to respond to the demand of affected areas, and the cost that each unit of shortage incurs on the HO is higher than the cost of procuring each item.

## 4. Models

Based on the defined notations and assumptions, the proposed model with a bidirectional option contract in the HSC is first presented and optimized in this section. Subsequently, to facilitate comparison with the proposed model, the benchmark and centralized models are also introduced and optimized.

### 4.1. Bidirectional option contract model

In this section, the decision-making models of the two supply chain members are examined, taking into account the bidirectional option contract. In the proposed model, at the beginning of the planning horizon, the supplier offers the HO a list of prices, including the wholesale price (w), the reservation price of the call option ${(o}_{c})$, the reservation price of the put option $\left(o_{p}\right)$, the exercise price of the call option ${(e}_{c})$, and the exercise price of the put option ${(e}_{p})$. Based on the proposed prices, the HO determines the quantity of items needed to be prepositioned in the pre-disaster phase $\left(Q_{BO}\right)$ and the quantity of items reserved for call options ${(q}_{c})$ and put options $\left(q_{p}\right)$. The full descriptions of these parameters and variables are provided in Table 2. When a disaster occurs during the planning horizon, the HO makes a decision regarding whether to exercise the call option, put option, or neither one based on the realized demand in the affected areas.

**Fig 2:**
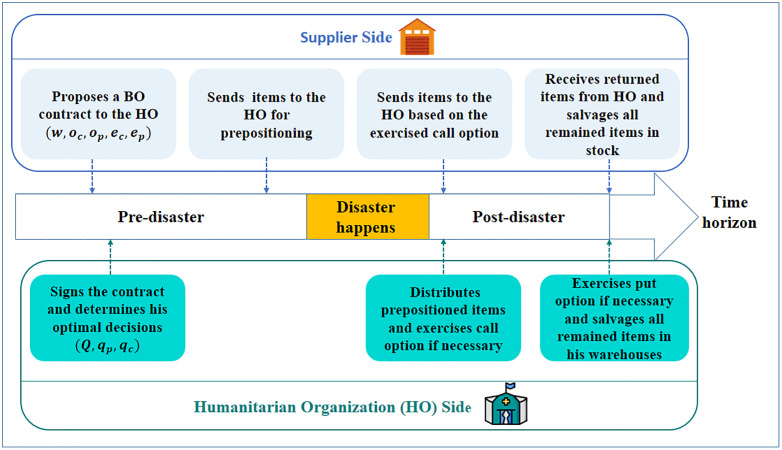
Decision sequences of supplier and humanitarian organization (HO), showing the order of decisions between the HO and the supplier.

The primary objective of the HO is to determine the optimal values of the wholesale, call, and put option orders $\left(Q_{BO},q_{c},q_{p}\right)$ while minimizing associated costs. On the other hand, the primary objective of the supplier is to determine the optimal reservation prices for the call and put options $\left(o_{c},o_{p}\right)$ while maximizing its total revenue. Given the nature of demand in HSC, the demand in this problem is considered stochastic, and a probability distribution function is used to cope with uncertainty. Fig 2 illustrates the structure of the studied HSC and the sequential decision-making process of the HO and supplier, highlighting the order of decisions that underlies the proposed model.

In the HO’s mathematical model with bidirectional option contract, when a disaster strikes, if the demand of affected areas (x) is less than the prepositioned quantity $\left(Q_{BO}\right)$ and $\left(Q_{BO}-x\leq q_{p}\right)$, the HO exercises $\left(Q_{BO}-x\right)$ units of put options and returns the difference to the supplier. If $\left(x\leq Q_{BO}\right)$ and $\left(Q_{BO}-x\geq q_{p}\right)$, the HO exercises $\left(q_{p}\right)$ units of put options and salvages $\left(Q_{BO}-x-q_{p}\right)$ units at a salvage value of $\left(v_{b}\right)$ per unit. If ($x\geq Q_{BO})$ and $\left(x-Q_{BO}\leq q_{c}\right)$, the HO exercises $\left(x-Q_{BO}\right)$ units of call option and receives $\left(x-Q_{BO}\right)$ units of relief items from the supplier. And if $\left(x\geq Q_{BO}\right)$ and $\left(x-Q_{BO}\geq q_{c}\right)$ the HO exercises all call options and will face a shortage of $\left(x-Q_{BO}-q_{c}\right)$.

If no disaster occurs throughout the duration of the contract, the HO exercises $\left(q_{p}\right)$ units of put options and returns ${(q}_{p})$ units of prepositioned items to the supplier and salvages the remaining $\left(Q_{BO}-q_{p}\right)$ units.

Therefore, the HO’s expected objective is as equation (1):


$$\begin{array}{cc} & E\left({TC}_{R}\left(Q_{BO},q_{p},q_{c}\right)\right)=\{wQ_{BO}+o_{c}q_{c}+o_{p}q_{p}-(1-\pi )\left[e_{p}q_{p}+v_{b}\left(Q_{BO}-q_{p}\right)\right]\\ & \;+\pi (E[-e_{p}\left[Min(q_{p},{\left(Q_{BO}-D\right)}^{+})]-v_{b}{\left(Q_{BO}-D-q_{p}\right)}^{+}+e_{c}[Min\left(q_{c},{\left(D-Q_{BO}\right)}^{+}\right)]\right]\\ & +g{\left(D-Q_{BO}-q_{c}\right)}^{+}])\} \end{array}$$ (1)

In the analysis above, the first term outlines the costs associated with purchasing wholesale orders to preposition items in the pre-disaster phase. The second and third terms calculate the costs of reserving call options and put options, respectively. The fourth term calculates the revenue generated from exercising the put options and the salvage value of the remaining items if no disaster occurs by the end of the contract period. Lastly, the fifth term estimates the costs of exercising put and call options in the event of a disaster.

Given the inherent uncertainty of demand and its probability density function, the expected costs incurred on the HO can be calculated as follows:


$$\begin{array}{cc} E\left({TC}_{R}\left(Q_{BO},q_{p},q_{c}\right)\right)= & (w-\pi g)Q_{BO}+\left(o_{c}-\pi g+\pi e_{c}\right)q_{c}+o_{p}q_{p}+\pi \mu g\\ & +(1-\pi )\left[-e_{p}q_{p}-\left(Q_{BO}-q_{p}\right)v_{b}\right]\\ & +\pi \{(e_{p}-v_{b})\int_{\text{0}}^{Q_{BO}-q_{p}}F(x)dx+(e_{c}-e_{p})\int_{\text{0}}^{Q_{BO}}F(x)dx+(g-e_{c})\int_{\text{0}}^{Q_{BO}+q_{c}}F(x)dx\} \end{array}$$ (2)

In Proposition 1, we derive the closed-form decisions for the HO based on a general distribution function for demand.

**Proposition 1:** The optimal values of the HO’s decisions are obtained from equations (3) to (5) as follows:


$$Q_{BO}=F^{-1}(\frac{\left(w+o_{p}-o_{c}-\pi e_{c}-(1-\pi )e_{p}\right)}{\pi (e_{p}-e_{c})}$$ (3)

$$q_{p}=Q-F^{-1}(\frac{o_{p}+(1-\pi )(v_{b}-e_{p})}{\pi (e_{p}-v_{b})}$$ (4)

$$q_{c}=F^{-1}(1+\frac{o_{c}}{\pi \left(e_{c}-g\right)})-Q$$ (5)

Proof of the Proposition 1 is presented in S1 Appendix.

**Corollary 1:** ${(Q}_{BO})$ has a negative relationship with ${(o}_{p})$, and positive relationships with ${(e}_{p})$, $\left(e_{c}\right)$ and ${(o}_{c})$. ${(q}_{p})$ has a negative relationship with ${(o}_{p})$, and positive relationships with ${(e}_{p})$, $\left(e_{c}\right)$ and ${(o}_{c})$. ${(q}_{c})$ has a positive relationship with ${(o}_{p})$, and negative relationships with ${(e}_{p})$, $\left(e_{c}\right)$ and ${(o}_{c})$.

See S2 Appendix for the proof of Corollary 1.

Corollary 1 is logical and consistent with real-world observations. In the real world, when a supplier increases the reservation price of each unit of the put option, HO’s tendency to preposition more items decreases. This is because when the per-unit reservation price of put options increases, the HO reserves fewer put options. Therefore, in order to reduce the risk of inventory surplus in case of low demand, the HO prepositions fewer items in its warehouses, and in order to reduce the risk of shortage when demand exceeds the reserved quantity, the HO increases the order quantity of call options. On the other hand, in case all parameters remain fixed and the supplier increases the price of exercising the put option, if no disaster occurs or the post-disaster demand is less than the prepositioned inventory, the HO’s income increases by each item that is returned to the supplier. Therefore, the HO tries to store more items before the disaster. As a result of this increase, since the likelihood of shortage decreases, the HO reduces the quantity of call options and, to mitigate inventory risk, increases the reservation of put options. Furthermore, if the price of reserving or exercising call options increases, the HO will incur higher costs in the event that demand exceeds the prepositioned inventory. Therefore, to reduce the risk of stock-outs, the HO stores more inventory in the pre-disaster phase and decreases the quantity of call options. On the other hand, to manage inventory risk, it increases the quantity of put options.

**Corollary 2:** The value of ${(Q}_{BO})$ is independent of (g) and ${(v}_{b})$. However, it has a negative relationship with (w) and a positive relationship with (π). The value of ${(q}_{p})$ is independent of (g), but it has negative relationships with (w) and ${(v}_{b})$, and it is not possible to comment on the relationship between the changes in ${(q}_{p})$ and (π). The value of ${(q}_{c})$ is independent of ${(v}_{b})$, but it has a positive relationship with (w) and (g), and it is not possible to comment on the relationship between ${(q}_{c})$ and (π).

See S3 Appendix for proof and analysis of Corollary 2.

In the supplier’s mathematical model with bidirectional option contract, the supplier proposes a set of five prices to the HO: wholesale price (w), call option reservation price $\left(o_{c}\right)$, put option reservation price $\left(o_{p}\right)$, call option exercising price $\left(e_{c}\right)$, and put option exercising price $\left(e_{p}\right)$. The wholesale price is already known at the time of the contract offer to the HO based on the market price. Moreover, the exercise prices of the call and put options based on assumptions 11 and 12 have specific values determined by the linear combination of their upper and lower bounds. Therefore, the supplier’s main concern is to determine the reservation prices of call and put options $\left(o_{c},o_{p}\right)$, in order to maximize its profit.

At the beginning of the contract period, the supplier sells some relief items at the wholesale price along with a number of call and put options to the HO. During the contract period, if a disaster occurs and the demand assessment (x) is less than the wholesale order ($Q_{BO}$) and $\left(Q_{BO}-x\leq q_{p}\right)$, the HO exercises $\left(Q_{BO}-x\right)$ units of put options and the supplier must pay $\left(e_{p}\right)$ per each returned item. Next, the supplier salvages all remaining items in its warehouses, including the items manufactured to respond to the call option exercised by the HO, and the items returned to the warehouses as a result of the exercise of the put options. If $\left(x\leq Q_{BO}\right)$ and $\left(Q_{BO}-x\geq q_{p}\right)$, the HO returns $q_{p}$ units of stock items in the pre-disaster phase. Moreover, the supplier pays $e_{p}q_{p}$ dollars to the HO and salvages all the returned items, in addition to the items it had manufactured to respond to the HO’s potential exercise of its call option. If $\left(x\geq Q_{BO}\right)$ and $\left(x-Q_{BO}\leq q_{c}\right)$, the supplier sends $\left(x-Q_{BO}\right)$ units of relief items to the HO after the latter exercises its call option. However, if $\left(x\geq Q_{BO}\right)$ and $\left(x-Q_{BO}\geq q_{c}\right)$, the supplier sells $q_{c}$ units to the HO and receives $e_{c}$ per each shipped item.

If no disasters occur during the contract period, the supplier receives $q_{p}$ units of items returned by the HO and salvages ($q_{p}+q_{c}$) items remaining in its warehouses.

The supplier’s expected profit can be obtained from equation (6) as follows:


$$\begin{array}{cc} E\left({TP}_{s}(o_{c},o_{p})\right)=\; & wQ_{BO}+o_{c}q_{c}+o_{p}q_{p}-c\left(Q_{BO}+q_{c}\right)-(\text{1}-\pi )\left[\left(e_{p}-v_{s}\right)q_{p}-v_{s}q_{c}\right]\\ & \;+\pi [\text{E}(\left(v_{s}-e_{p}\right)Min\left(q_{p},{\left(Q_{BO}-D\right)}^{+}\right)+v_{s}(Min(\left(q_{c},{\left(Q_{BO}+q_{c}-D\right)}^{+}\right)\\ & \;+e_{c}Min\left(\left(q_{c},{\left(Q_{BO}-Q\right)}^{+}\right)\right)] \end{array}$$ (6)

Considering the probability distribution function for demand, the supplier’s profit can be reformulated as follows:


$$\begin{array}{cc} E\left({TP}_{s}(o_{c},o_{p})\right)= & wQ_{BO}+o_{c}q_{c}+o_{p}q_{p}-c\left(Q_{BO}+q_{c}\right)-(1-\pi )\left[\left(e_{p}-v_{s}\right)q_{p}-v_{s}q_{c}\right]\\ & +\pi [-e_{p}\int_{Q_{BO}-q_{p}}^{Q_{BO}}F(x)d(x)+e_{c}(q_{c}-\int_{Q_{BO}}^{Q_{BO}+q_{c}}F(x)d(x))+v_{s}\int_{Q_{BO}-q_{p}}^{Q_{BO}+q_{c}}F(x)d(x)] \end{array}$$ (7)

In the profit function above, the first term calculates the supplier’s revenue from selling relief items at a wholesale price for prepositioning in the HO’s warehouses. The second and third terms calculate the revenue from call and put option reservations, respectively. The fourth term calculates the total production cost. The fifth term calculates the salvage value of the items remaining in the supplier’s inventory due to the non-exercise of call and exercise of put options when no disaster occurs during the contract period. And the last term calculates the cost and revenue resulting from exercising the call and put options or the salvage value of the remaining items in proportion to the demand generated by the occurrence of a disaster.

Proposition 2: The optimal values of the supplier’s decisions are obtained from equations (8) and (9) as follows:


$$\begin{array}{cc} o_{c}= & (e_{c}-g)(\overset{\text{2}}{\underset{b}{v}}\left(g-v_{s}+c-e_{c}\right)-e_{p}v_{s}(g+c)+\overset{\text{2}}{\underset{s}{v}}\left(e_{p}-e_{c}\right)+\left(ce_{c}+w\left(g-e_{c}\right)\right)\left(e_{p}-2v_{b}+v_{s}\right)\\ & +2v_{b}e_{c}v_{s})/(\overset{\text{2}}{\underset{b}{v}}\left(e_{c}-2g+v_{s}\right)+g^{\text{2}}\left(2v_{b}-v_{s}-e_{p}\right)+2v_{s}\left(e_{p}g-e_{c}v_{b}\right)+\overset{\text{2}}{\underset{s}{v}}\left(e_{c}-e_{p}\right)) \end{array}$$ (8)

$$\begin{array}{cc} {\text{o}}_{\text{p}}= & {\left({\text{e}}_{\text{p}}-{\text{v}}_{\text{b}}\right)}^{\text{2}}(\text{c}\left({\text{e}}_{\text{c}}-\text{g}\right)-{\text{g}}^{\text{2}}-\text{w}\left({\text{e}}_{\text{c}}+{\text{v}}_{\text{s}}\right)+\text{g}\left({\text{v}}_{\text{s}}+\text{2w}\right))/(\overset{\text{2}}{\underset{\text{b}}{\text{v}}}\left({\text{e}}_{\text{c}}-\text{2g}+{\text{v}}_{\text{s}}\right)+{\text{g}}^{\text{2}}\left(\text{2}{\text{v}}_{\text{b}}-{\text{v}}_{\text{s}}-{\text{e}}_{\text{p}}\right)\\ & +\text{2}{\text{v}}_{\text{s}}\left({\text{e}}_{\text{p}}\text{g}-{\text{e}}_{\text{c}}{\text{v}}_{\text{b}}\right)+\overset{\text{2}}{\underset{\text{s}}{\text{v}}}\left({\text{e}}_{\text{c}}-{\text{e}}_{\text{p}}\right)) \end{array}$$ (9)

Where by substituting $e_{p}$ and $e_{c}$, according to formulas ${(e}_{c}=\lambda w+g(1-\lambda ))$ and $\left(e_{p}=\lambda v_{b}+w(1-\lambda )\right)$, we have:


$$\begin{array}{cc} o_{c}= & (\lambda (w-g)(\overset{\text{2}}{\underset{s}{v}}\left(\lambda \left(v_{b}+g-2w\right)+w-g\right)-(g+c)v_{s}\left(w+\lambda \left(v_{b}-w\right)\right)+\overset{\text{2}}{\underset{b}{v}}\left(c+v_{s}+\lambda (g-w)\right)\\ & +2v_{b}v_{s}\left(g-\lambda (g-w)\right)-\left(w+v_{s}-2v_{b}+\lambda \left(v_{b}-w\right)\right)\left(cg(\lambda -1)+\lambda w(w-g-c)\right))/\lambda (w-g)(\overset{\text{2}}{\underset{s}{v}}\\ & +\overset{\text{2}}{\underset{b}{v}})+\lambda (w-v_{b})(\overset{\text{2}}{\underset{s}{v}}+g^{\text{2}})+\overset{\text{2}}{\underset{s}{v}}(g-w)-g^{\text{2}}(v_{s}+w-2v_{b})+\overset{\text{2}}{\underset{b}{v}}(v_{s}-g)-2\lambda v_{b}v_{s}(w-g)\\ & -g(2v_{b}v_{s}+2w(\lambda -1)v_{s}) \end{array}$$ (10)

$$\begin{array}{cc} o_{p}= & \left((\lambda -1{)}^{\text{2}}(g-w){\left(v_{b}-w\right)}^{\text{2}}\left(\lambda (w-c)+v_{s}-g\right))/\lambda (w-g)(\overset{\text{2}}{\underset{s}{v}}+\overset{\text{2}}{\underset{b}{v}})+\lambda (w-v_{b})(\overset{\text{2}}{\underset{s}{v}}+g^{\text{2}}\right)\\ & +\overset{\text{2}}{\underset{s}{v}}(g-w)-g^{\text{2}}(v_{s}+w-2v_{b})+\overset{\text{2}}{\underset{b}{v}}(v_{s}-g)-2\lambda v_{b}v_{s}(w-g)-g(2v_{b}v_{s}+2w(\lambda -1)v_{s}) \end{array}$$ (11)

Proof of the Proposition 2 is presented in S4 Appendix.

**Corollary 3:** When $\left(v_{s}=v_{b}\right)$, the quantity of the put option order is zero and a bidirectional option contract is converted into a call option contract.

See S5 Appendix for proof of Corollary 3.

Corollary 3 is also consistent with the real world. This is because in cases where the salvage values of items are equal for the supplier and the HO, it is more profitable for the supplier if the HO salvages the items remaining in its own warehouses. Therefore, the supplier maximizes the reservation price of put options. As a result of this decision, the HO sets its reserved option value to zero, practically rendering the bidirectional option contract a one-way call option contract, as evidenced by Corollary 3. Furthermore, to compare and demonstrate the performance of the proposed model, a decentralized model – featuring a wholesale contract – and a centralized model are presented in the following subsections.

**Corollary 4:** The optimal quantities of call and put option reservation prices are independent of both the probability of the occurrence of the disaster (π) and the maximum demand (b), provided that the demand follows a uniform distribution.

**Proof:** Assuming the demand distribution function follows a uniform distribution, since $\frac{\partial o_{p}}{\partial \pi }=\frac{\partial o_{c}}{\partial \pi }=\frac{\partial o_{p}}{\partial b}=\frac{\partial o_{c}}{\partial b}=0$, the optimal reservation prices for the call and put options are independent of both the disaster occurrence probability and the maximum demand.

Considering a uniform probability distribution function for demand, the closed‐form expression for the supplier’s optimal contract price does not include either the disaster occurrence probability or the maximum demand. This is despite the fact that both parameters have a significant influence on the HO’s optimal decisions and directly affect its order quantity. Mathematically, this outcome arises because, under a uniform distribution, these parameters appear only as multiplicative scaling factors in the HO’s best‐response function and do not affect the slope of the price–quantity relationship that is relevant for the supplier. When this reaction function is substituted into the supplier’s optimization problem, such scaling factors cancel out in the first‐order condition, leading to an optimal price that is independent of these parameters. From an economic perspective, this implies that in a Stackelberg setting with the supplier as leader, the optimal pricing decision depends solely on the shape (price elasticity) of the HO’s demand function, not on its scale. Variations in disaster probability or maximum demand shift the overall market size and the supplier’s profit level, but do not alter the optimal contract price.

### 4.2. Benchmark model based on wholesale price contract

In order to evaluate the performance of the proposed model, we use a wholesale contract as the benchmark model. In the proposed model, the supply chain consists of an HO and a supplier. Each member independently optimizes its objectives regardless of the other member’s objectives. Prior to the planning period, the HO orders ${(Q}_{w})$ units of relief times at the wholesale price (w) proposed by the supplier. At the start of the planning period, the HO receives the items from the supplier and stores them in its warehouses. If a disaster occurs during the planning period, the incoming demand is satisfied by the items readily available in the warehouses. If the demand is less than the quantity of stored items, the remaining items are salvaged at the end of the period. If, however, the demand exceeds the quantity of stored items, a portion of the demand is satisfied, and the rest is deemed as shortage. If no disaster occurs throughout the planning period (i.e., until the items expire), all remaining items are salvaged at a value of ${(v}_{b})$.

Based on the description above, the objective function of the HO can be expressed in the form of equation (12) as follows:


$$E\left({TC}_{R}\left(Q_{w}\right)\right)=wQ_{w}-(1-\pi )\left[v_{b}Q_{w}\right]+\pi (E\left[v_{b}{\left(Q_{w}-D\right)}^{+}+g{\left(D{-Q}_{w}\right)}^{+}\right])$$ (12)

The first term of the objective function calculates the procurement cost of the ordered items based on the wholesale price. The second term represents the salvage value of the prepositioned items until the end of the planning period, provided no disaster occurs. Lastly, the third term calculates the salvage value of the remaining inventory and shortage cost, based on realized demand, in the event of a disaster.

Considering a probability distribution function for demand, the equation above is reformulated as follows:


$$E\left({TC}_{R}\left(Q_{w}\right)\right)=wQ_{w}-(1-\pi )\left[v_{b}Q_{w}\right]+\pi (\int_{\text{0}}^{Q_{w}}-v_{b}(Q_{w}-x)f(x)dx+\int_{Q_{w}}^{+\infty }g({x-Q}_{w})f(x)dx)$$ (13)

**Proposition 3:** The optimal quantity of the HO’s prepositioned items can be obtained from equation (14) as follows:


$$Q_{w}=F^{-1}(1+\frac{v_{b}-w}{\pi \left(g-v_{b}\right)})$$ (14)

The proof of the Proposition 3 is presented in S6 Appendix.

**Corollary 4:** The optimal quantity of the HO’s prepositioned items in the benchmark model has positive relationships with ${(v}_{b})$ and (g), and a negative relationship with (w).

See S7 Appendix for proof of Corollary 4. Corollary 4 is logical because if the wholesale price increases, the HO will purchase fewer relief items. The opposite is also true, given that if the shortage cost or salvage value increases, the HO purchases more items.

Based on the HO’s decision, the supplier’s objective function can be expressed as equation (15):


$${TP}_{s}\left(Q_{w}\right)=(w-c)Q_{w}$$ (15)

Considering the optimal decision of the HO and assuming that demand is uniformly distributed in the interval [0,b], the supplier’s profit can be calculated as equation (16):


$${TP}_{s}\left(Q_{w}\right)=(w-c)(b+\frac{b(v_{b}-w)}{\pi \left(g-v_{b}\right)})$$ (16)

### 4.3. Centralized system

In centralized mode, there is a general supply chain in which a chain manager optimizes the total profit of the supply chain without considering the profit of each member individually. Considering the order quantity $Q_{cs}$, the expected objective function of the centralized model can be obtained as equation (17):


$$E({TP}_{cs}\left(Q_{cs})\right)=-cQ_{cs}+(1-\pi )\left[v_{s}Q_{cs}\right]+\pi (E\left[v_{s}{\left(Q_{cs}-D\right)}^{+}-g{\left(D{-Q}_{cs}\right)}^{+}\right])$$ (17)

The first term in the function above calculates production costs. The second term calculates the salvage value of the manufactured items in the event that no disaster occurs. And the last term determines the salvage value and shortage cost in the event of a disaster.

Considering the stochastic distribution function of demand, the function above can be reformulated as follows:


$$E({TP}_{cs}\left(Q_{cs})\right)=-cQ_{cs}+(1-\pi )\left[v_{s}Q_{cs}\right]+\pi (v_{s}\int_{\text{0}}^{Q_{cs}}F(x)dx+gQ_{cs}-g\mu -g\int_{\text{0}}^{Q_{cs}}F(x)dx)$$ (18)

**Proposition 4:** the optimal production quantity in the centralized system is obtained as equation (19):


$$Q_{cs}=F^{-1}(\frac{c-(1-\pi )v_{s}-\pi g}{\pi \left(v_{s}-g\right)})$$ (19)

The proof of Proposition 4 is included in S8 Appendix.

**Corollary 5:** ${(Q}_{cs})$ has a negative relationship with (c) and positive relationships with (g), $\left(v_{s}\right)$ and (π).

See S9 Appendix for proof of Corollary 5.

Corollary 5 is entirely logical because, as the per-unit production cost increases, the supply chain decision-maker will attempt to reduce the order quantity. As the occurrence probability of a disaster increases, the production level in the supply chain also increases to reduce the risk of a supply shortage. In addition, as the per-unit shortage cost increases, the order quantity also increases, imposing lower costs on the supply chain. Similarly, as the per-unit salvage value increases, larger quantities of relief items are stored in warehouses to reduce the risk of a supply shortage.

## 5. Computational results and sensitivity analysis

This section examines the impact of changes in the value of key parameters on the decisions of supply chain members in scenarios that involve the centralized system, the bidirectional contract, or the wholesale contract. To ensure reliable results, we set the baseline parameter values for natural disasters, including earthquakes, inspired by the ranges used in Patra and Jha [19] and Yang and Wu [48]. Consequently, the following values have been assigned to the parameters: $v_{b}=\$30$, $v_{s}=\$45$, c=$50, w=$70, g=$150 π=0.9, and b=500. When determining these values, the logical and realistic relationships between the parameters stated in the Problem Description Section and the intervals proposed in the relevant literature by our peers were meticulously considered. For instance, in the real world, the salvage value of each product is higher for the supplier compared to the HO. Hence, the supplier’s salvage value in our study is set at $45, with the HO’s salvage value set at $30. Furthermore, in the case of shortage, the HO must acquire the necessary relief items immediately from the spot market, resulting in significantly higher per-unit shortage cost than the wholesale price. Consequently, we have set the shortage cost at $150 and the wholesale price at $70.

As part of the optimization analysis, for λ = 0.5, Table 3 presents the objective function values of the HO, the supplier, and the overall supply chain under three settings: the wholesale contract (benchmark model), the proposed bidirectional option contract, and the centralized model. As observed in Table 3, when the bidirectional option contract is applied instead of the wholesale contract, the objectives of the HO and the supplier improve by 4.18% and 39%, respectively, while the total supply chain cost is reduced by an average of 20%. Furthermore, under the wholesale contract, the overall performance remains approximately 33% away from the centralized model, whereas with the bidirectional option contract, this gap is reduced to only 5%. These results highlight the superiority of the proposed model based on the bidirectional option contract in enhancing the objectives of supply chain members and bringing the system closer to the centralized model.

**Table 3:** Optimization analysis (objective function comparison for the HO, supplier, and centralized system under different contracts).

Decision maker	Contract type			Improvement over wholesale contract (%)
	Wholesale contract	Bidirectional contract	Centralized system	
**HO cost**	23046.3	22081.6	–	4.18%
**Supplier profit**	6296.3	8791.98	–	39%
**Total cost**	16750	13289.62	12558	20%
**Distance from centralized (%)**	33%	5%	–	–

Based on the results obtained from the optimality analysis and the comparison of the bidirectional option contract model with the wholesale and centralized models in the proposed HSC, as reported in previous studies (e.g., [18,19]), the bidirectional option contract outperforms the wholesale contract and can lead to improved coordination among supply chain members. Unlike prior studies, which focused solely on optimizing the HO’s decisions, our study simultaneously derives the optimal decisions for both the HO and the supplier. Furthermore, more realistic assumptions reflecting practical settings are incorporated, including differentiated salvage values for each member, varying quantities of call and put options, and distinct reservation and exercise prices for both types of options. Therefore, our results not only confirm the effectiveness of bidirectional option contracts in mitigating risk under disaster conditions, but also provide a more comprehensive perspective on contract design in such supply chains, capturing practical aspects that were not addressed in earlier analyses.

To perform sensitivity analysis, the value of each parameter has been altered within its feasible ranges while keeping the other parameters at their baseline values. Table 4 shows the changes in the decisions of the members for different values of λ in the range of (0 < λ < 1). Here, similar to the study by Yang and Wu [48], Δ is defined as a parameter to measure the improvements caused by bidirectional option contracts in the total supply chain cost compared to the wholesale price contract and can be obtained as follows:

**Table 4:** Effects of changing parameter λ on decisions and objective functions of chain members in different models.

λ	W				CS		BO								Δ
	$Q_{W}$	HO Cost	Supplier Profit	Total Cost	$Q_{CS}$	Total Cost	$o_{c}$	$o_{p}$	$Q_{BO}$	$q_{c}$	$q_{p}$	HO Cost	Supplier Profit	Total Cost	
0.1	314	23046.3	6296.3	16750	473	12558.9	0.51	18.17	341	122	116	22574	8987.1	13586.9	75%
0.2							1.3	15.17	331	123	123	22417.1	8930.04	13487.06	77%
0.3							2.37	12.34	320	124	131	22281	8877.63	13403.37	79%
0.4							3.73	9.7	309	125	140	22167.9	8831.02	13336.88	81%
0.5							5.4	7.26	297	127	151	22081.6	8791.98	13289.62	82%
0.6							7.39	5.06	285	129	164	22028	8763.07	13264.93	83%
0.7							9.75	3.13	271	131	181	22017	8748.13	13268.87	83%
0.8							12.51	1.56	256	134	203	22068	8753.24	13314.76	81%
0.9							15.78	0.44	239	139	232	22217	8788.61	13428.39	79%

(W = Wholesale contract, CS = Centralized system, BO = Bidirectional option contract).


$$\Delta =\frac{CostinBO-CostinW}{CostinCS-CostinW}\times 100$$ (20)

Where Cost represents the total supply chain cost, *BO* represents the bidirectional option contract mode, *W* represents the wholesale price contract mode, and *CS* represents the centralized mode. In terms of impact on costs, as shown in Table 4, for different values of λ, the total supply chain cost is lower in the case where the bidirectional option contract is implemented compared to the wholesale contract. This clearly indicates that for different λ values in the [0, 1] range, the bidirectional option contract is superior to the wholesale price contract as it can achieve a win-win solution by ensuring efficient coordination between the two supply chain members. According to the values reported in Table 4, the maximum cost savings (83%) for the entire supply chain are achieved when λ = 0.6 and λ = 0.7.

The value of *λ* significantly impacts the supplier’s pricing decisions for call and put options. As *λ* increases, exercise prices for both options decrease. In response, the supplier strategically adjusts its reservation prices, increasing the call option’s reservation price to maintain profit and decreasing the put option’s reservation price to motivate the HO for bilateral cooperation. See Table 4 for detailed results.

As for the potential impact of changes in the value of *λ* on the HO’s decisions, it should be said that as λ increases, the exercise price of the call option almost reaches the wholesale price. This incentivizes the HO to reduce wholesale orders but increase call option purchases to mitigate potential shortage risks. In contrast, raising the value of λ and reducing the reservation price of the put option encourages the HO to increase its put option orders to cope with the risk of surplus inventory. See Table 4 for detailed results.

Table 5 displays the changes in the optimal decisions of the HO for different values of π, which represents the disaster occurrence probability, when λ is set to 0.5. As the occurrence probability of a disaster, namely a major earthquake, increases in a particular area, the HO increases the quantity of its prepositioned orders to mitigate the risk of supply shortage. On the other hand, it reduces the quantity of the reserved put options and call options. Conversely, if the occurrence probability of an earthquake is deemed low, the HO aims to reduce the initial order quantity and increase the reservation of call options to mitigate potential shortage risks. Additionally, the HO will consider raising the quantity of put options to be fully prepared for potential inventory risks. These results are detailed in Table 5.

**Table 5:** Impacts of changing in probability of disaster occurrence (π) on HO’s decisions.

Decision variables HO	The probability occurrence of a disaster						
	π=0.65\)	π=0.7	π=0.75	π=0.8	π=0.85	π=0.9	π=0.95
$Q_{BO}$	219	239	257	272	286	297	308
$q_{p}$	209	194	181	170	160	151	143
$q_{c}$	176	163	153	143	135	127	121

**Fig 3:**
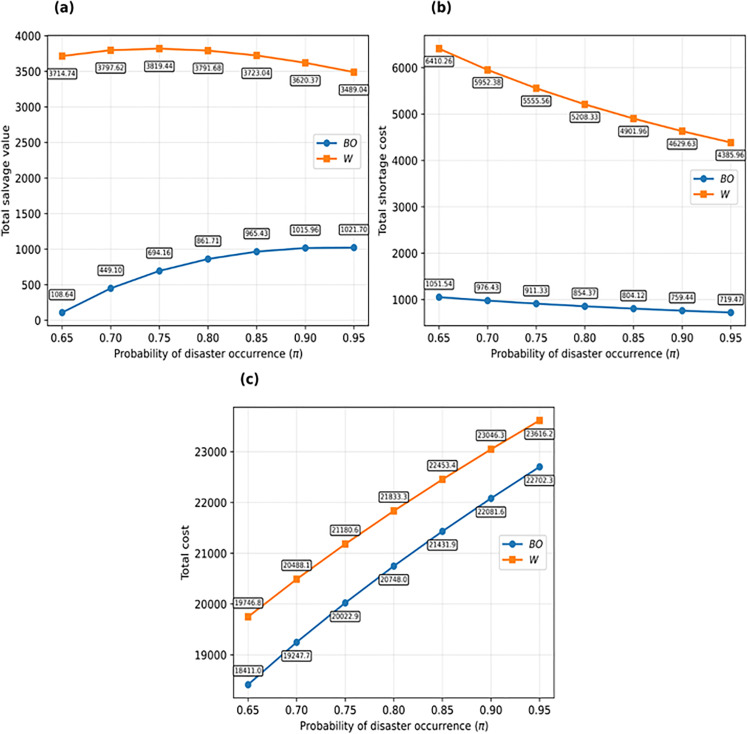
Impact of the probability of disaster occurrence (π) on: (a) total salvage value, (b) total shortage cost and (c) total cost under BO and W models.

In Fig 3, the impact of the disaster occurrence probability parameter on the total cost of the HO, the shortage cost of the HO, and the total salvage value of the remaining goods for the HO is evaluated under the BO and W models. As shown in the figures, under the bidirectional option contract—comprising both a call option for additional purchases and a put option for returning surplus goods—the total cost and shortage cost are consistently lower than those in the wholesale contract. This is primarily due to the call option’s ability to reserve additional quantities when needed, which helps control shortages and associated costs, thereby highlighting the superiority of the bidirectional option contract. Furthermore, the salvage value under the bidirectional option contract is lower than that under the wholesale contract, as the put option enables the return of unsold goods to the supplier, further demonstrating the advantage of the bidirectional option contract over the wholesale contract.

**Fig 4:**
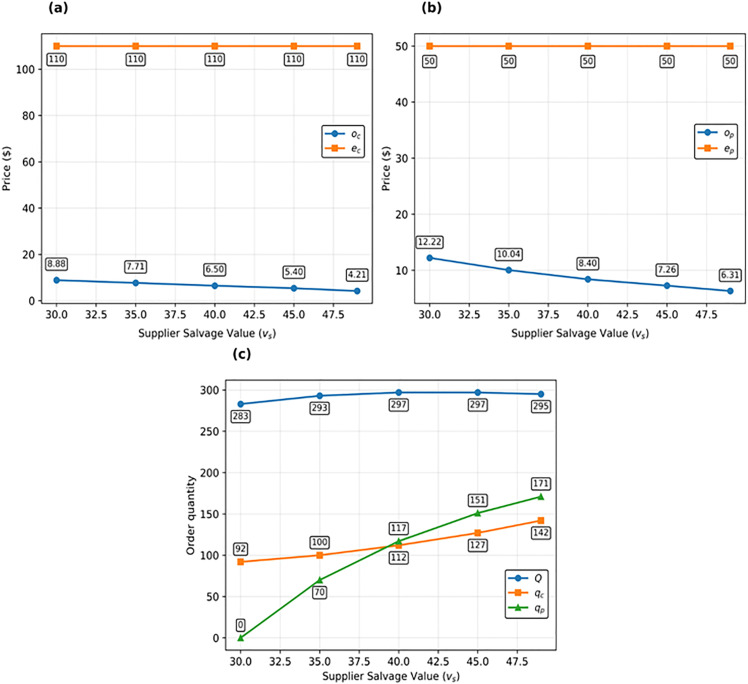
Impact of supplier’s salvage ($\begin{array}{c} v_{s} \end{array}$) value on decisions of HO and supplier: (a) on call option prices ($\begin{array}{c} o_{c} \end{array}$ and $\begin{array}{c} e_{c} \end{array}$), (b) on put option prices ($\begin{array}{c} o_{p} \end{array}$ and *e_p_*), and (c) on HO’s decisions ($\begin{array}{c} Q_{BO},q_{p}, \end{array}$ and $\begin{array}{c} q_{c} \end{array}$).

In Fig 4, the impact of changes in the salvage value of each product for the supplier at the end of the contract period on the decision variables of the supplier and HO is investigated. As can be seen, when the supplier salvage value increases and the other parameters remain fixed, the supplier’s willingness to motivate the HO to reserve call and put options increases. This is because if the items are returned by the HO (through the put option) or if the call option is not exercised, the supplier will gain more profit per each item remaining in its warehouses. Therefore, the supplier reduces the price of the reservation to encourage the HO to reserve more options. As a result, the value of the options reserved by the HO increases, and, as Fig 4 shows, the slope of the graph for the put option is greater than that of the call option. Changes in the supplier’s salvage value do not significantly influence the optimal initial order. This implies a low degree of sensitivity to this parameter. Another noteworthy observation, evident from Fig 4.c. and parametrically supported by Corollary 3, is that when the supplier salvage value equals the HO’s ($v_{s}=v_{b}=30)$, the amount reserved for put options becomes zero, effectively converting the bidirectional option contract into a call option contract. However, as the supplier’s salvage value increases, the significance of the put option contract within the bidirectional option contract becomes more pronounced.

**Fig 5:**
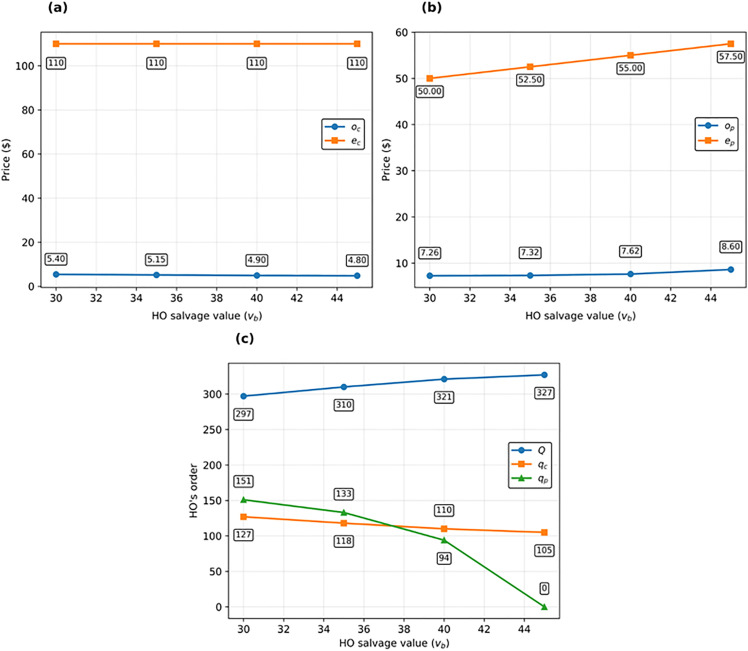
Impact of HO’s salvage values ($\begin{array}{c} v_{b}) \end{array}$ on decisions of HO and supplier: (a) on call option prices ($\begin{array}{c} o_{c} \end{array}$ and $\begin{array}{c} e_{c} \end{array}$), (b) on put option prices $\begin{array}{c} (o_{p} \end{array}$ and $\begin{array}{c} e_{p} \end{array}$), and (c) on HO’s decision ($\begin{array}{c} Q,q_{p}, \end{array}$ and $\begin{array}{c} q_{c} \end{array}$).

In Fig 5, the impact of changes in the salvage value of each item by the HO is investigated. An increase in $\left(v_{b}\right)$ leads to an increase in the price of exercising each unit of the put option; consequently, the supplier increases the reservation price for each unit of the put option to compensate for potential costs. On the other hand, the HO is less cautious about wholesale orders and increases the quantity of its wholesale orders. Hence, with the probability of disaster occurrence remaining fixed, the quantity of reserved call options decreases. As a result of this change, the supplier reduces the reservation price of call options to take control of its profit and maintain the HO’s tendency to reserve call options.

**Fig 6:**
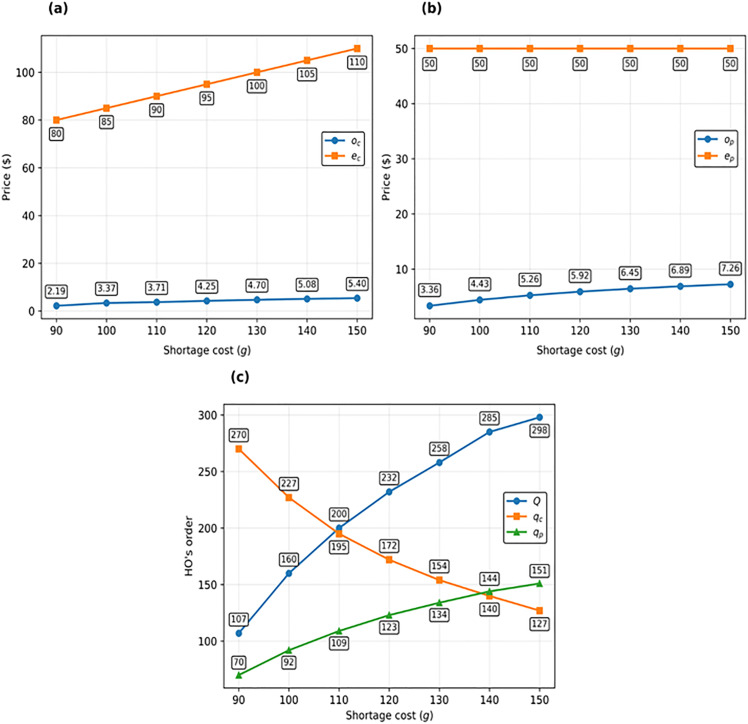
Impact of shortage cost ($\begin{array}{c} g \end{array}$) on decisions of HO and supplier: (a) on call option prices $\begin{array}{c} {(o}_{c} \end{array}$ and $\begin{array}{c} e_{c}) \end{array}$, (b) on put option prices $\begin{array}{c} (o_{p} \end{array}$ and $\begin{array}{c} e_{p} \end{array}$), and (c) on the HO’s decisions ($\begin{array}{c} Q,q_{p}, \end{array}$ and $\begin{array}{c} q_{c} \end{array}$).

Fig 6 demonstrates the impact of changes in the cost of shortages on the decisions of the supply chain members. Evidently, as the shortage cost increases, the exercise price increases as well. As a result, the HO stores more items in its inventory and reserves fewer call options. Thus, the supplier increases the reservation price of each call option in order to increase its profit. With a rise in the quantity of wholesale orders, the quantity of put options reserved by the HO is inevitably raised to be better equipped for coping with inventory risks. Therefore, in order to achieve greater profit, the supplier increases the reservation price of each put option. As depicted in Fig 6, the increase in the reservation price of the put option is greater than that of the call option; this is because the HO has a stronger incentive to reserve put options than call options when the shortage cost increases. These results are detailed in Fig 6.

## 6. Managerial implications

In an HSC, due to the inherent uncertainty of disaster occurrence and the resulting demand, if HOs employ prepositioning as a pre-disaster strategy for storing relief items, they may face inventory or shortage risks. One way for HOs to improve their flexibility and minimize risks is by establishing a bidirectional option contract with their supplier(s). On this basis, in the present study, we provide the following insights and practical recommendations, which should be considered by HOs and suppliers before signing such a contract:

- In an HSC, using a bidirectional option contract is most often more profitable for both members of the supply chain than using a wholesale contract. In such supply chains, if the supplier lowers its exercise prices, it reduces the cost of reserving put options and increases the cost of reserving call options, thereby generating more profit than it can gain from a wholesale contract. The HO can also reduce its wholesale orders and increase its reserved orders to minimize costs and manage inventory and shortage risks by changing the reservation prices and exercising its call options as stated above. Therefore, a bidirectional contract can lead to coordination among the chain members. From a managerial perspective, suppliers can maximize their profit by setting call and put prices strategically, while HOs can reduce inventory and shortage risks by adjusting their orders accordingly.
- The number of call and put option orders reserved by the HO directly depends on the number of its wholesale orders. The more wholesale orders are placed, the more put options are secured to reduce inventory risks. Unlike the FEMA case [9], where the HO faced inventory risk due to the lack of put option authority, implementing a bidirectional option contract allows HOs to better manage inventory. Furthermore, if the number of wholesale orders decreases to mitigate shortage risks, the number of reserved call options increases; in contrast, in the Wenchuan Earthquake case [8], the HO encountered shortage risks due to insufficient items in the warehouses. These observations indicate the high flexibility and risk management advantages created by implementing a bidirectional option contract for the HO in real-world disaster response scenarios. Therefore, managers at HOs should carefully plan the balance between wholesale orders and reserved call/put options. By aligning the number of options with wholesale orders, they can reduce inventory risks and avoid shortages, while maintaining high operational flexibility in disaster response.
- The reservation prices for call and put options proposed by the supplier, if demand follows a uniform distribution, are completely independent of both the probability of disaster occurrence and the demand distribution. However, the quantity of orders placed by the HO is directly affected by the probability of disaster occurrence and the demand distribution. For example, the higher the probability of a disaster, the more items the HO tends to store in its warehouses during the pre-disaster phase, while the number of reserved call and put options decreases. Managers at HOs should adjust their orders based on both the probability of disaster occurrence in each region and the corresponding demand distribution. This approach ensures that sufficient inventory is prepositioned in high-risk areas while avoiding unnecessary costs in low-risk areas. By optimizing the use of call and put options, managers can maintain operational flexibility and minimize resource waste. Furthermore, decision-making should be driven by reliable data on disaster probabilities and demand patterns to ensure that ordering and reservation strategies are both accurate and cost-effective.
- If the salvage value of items is the same for both the supplier and the HO, the bidirectional option contract transforms into a call option contract. This outcome is completely consistent with real-world conditions. When this situation occurs, it is no longer profitable for HOs to reserve put options, and the HOs make their decisions regarding call options and wholesale orders only in accordance with the nature of the problem. When the salvage value of items is the same for both the supplier and the HO, managers at HOs should focus solely on call options and wholesale orders, as reserving put options is no longer profitable. This simplification allows for more straightforward decision-making based on the actual needs and nature of the problem, while reducing unnecessary costs. Additionally, managers can better coordinate with suppliers and maintain operational flexibility under this simplified contract structure.

Overall, implementing bidirectional option contracts and managing orders strategically allows HOs to reduce inventory risks, avoid unnecessary costs, and maintain high operational flexibility in disaster response. HO managers should align their call and put options with wholesale orders and consider various real-world factors, including the probability of disaster occurrence and demand distribution, in their decision-making. Additionally, when the salvage value of items is equal, they can simplify decisions by focusing solely on call options. On the supplier side, managers can maximize profits by strategically setting call and put prices, improving coordination with HOs, and streamlining processes when the contract reduces to a call-only structure. This integrated approach enhances decision-making, strengthens supply chain coordination, and improves overall efficiency.

## 7. Conclusion and future research

Various natural and man-made disasters, typified by the recent COVID-19 pandemic, occur worldwide every year and affect large populations. In such circumstances, the management of HSCs plays a key role in preserving the lives and livelihood of individuals. In such supply chains, due to the high uncertainty of disaster occurrence and the resulting demand spike, chain members often face serious challenges when it comes to making decisions. A relatively novel solution to this problem is the use of bidirectional option contracts.

In this study, the impact of implementing a bidirectional option contract in an HSC with realistic assumptions, such as taking into account the likelihood of a disaster occurrence was examined. Importantly, the problem was modeled from the perspective of both chain members, i.e., the supplier and the HO. The HO’s optimal decisions, including the quantity of wholesale orders, reserved call options, and reserved put options, were extracted by considering a general distribution function for demand. Moreover, the supplier’s optimal decisions, including the reservation prices of call and put options were also reported. In order to evaluate the proposed model, its performance was compared to a benchmark model (the wholesale contract model) and a centralized model, and the optimal decisions in each case were extracted.

From a numerical perspective, the bidirectional option contract demonstrated superior performance compared to the wholesale contract, with cost savings of up to 83% observed in the total supply chain cost, particularly when the disaster probability λ ranged between 0.6 and 0.7. These results confirm the model’s potential to improve coordination and reduce operational costs, although the exact benefits depend on the scenario-specific parameters. From a managerial standpoint, bidirectional option contracts offer a superior alternative to wholesale contracts in terms of profitability and risk-sharing. The supplier can manipulate the reservation prices of call and put options to create incentives, while the HO can adjust its ordering strategy to reduce costs and minimize risks, enhancing coordination across the supply chain.

The HO’s flexibility is further strengthened by the ability to adjust the mix between wholesale orders and reserved options; for example, when the HO increases wholesale orders, it also tends to reserve more put options to mitigate the risk of surplus inventory. While the supplier’s reservation prices are independent of disaster probabilities, the HO’s decisions are highly sensitive to these probabilities, and in high-risk regions, the HO prefers to stock more items pre-disaster and reduce reliance on options, demonstrating adaptive preparedness behavior. Finally, when the salvage value is equal for both parties, the bidirectional option contract effectively behaves like a standard call option contract, making the reservation of put options less beneficial, which aligns with observed real-world practices. These insights suggest that decision-makers in humanitarian operations should consider adopting bidirectional option contracts, especially in volatile environments, to improve preparedness, reduce risk, and enhance coordination with suppliers.

This study has a few limitations that open avenues for future research. First, supply chain members were assumed to be risk-neutral, whereas in practice humanitarian organizations and suppliers often exhibit risk-averse or loss-averse behavior (e.g., [53,54]). Extending the model to incorporate such risk preferences could improve its realism. Second, practical constraints such as budget restrictions and logistical limitations were not explicitly modeled, although they strongly influence decision-making in humanitarian operations. Future research could address this gap by integrating these constraints into the analysis (e.g., [55]). Third, the study relied on the availability of sufficient reliable historical data to characterize uncertainty. In many humanitarian settings, however, such data may be scarce. In these cases, robust optimization approaches offer a promising alternative to probability-based methods for handling uncertainty (e.g., [41,56]).

## Supporting information

S1 Appendix Proof of Proposition 1. (DOCX)

S2 Appendix Proof of corollary 1. (DOCX)

S3 Appendix Proof of corollary 2. (DOCX)

S4 Appendix Proof of Proposition 2. (DOCX)

S5 Appendix Proof of corollary 3. (DOCX)

S6 Appendix Proof of Proposition 3. (DOCX)

S7 Appendix Proof of corollary 4. (DOCX)

S8 Appendix Proof of Proposition 4. (DOCX)

S9 Appendix Proof of Corollary 5. (DOCX)
