# Supplementary material for: Pricing and procurement strategies in the relief supply chain via bidirectional option contract
Source: PLoS One. 2026 Apr 1;21(4):e0341427. doi: 10.1371/journal.pone.0341427 (PMC13042840; doi:10.1371/journal.pone.0341427)
Supplement: S1 Appendix — (DOCX) [file pone.0341427.s001.docx]

**S1 Appendix. Proof of Proposition 1**

Since the HO's objective function is nonlinear, we need to find the critical points of the function with the first derivative of the function with respect to each decision variable to determine the optimal values of the HO's decisions.

| $\frac{\partial E({TC}_{R}(Q_{BO},q_{p},q_{c})}{\partial Q_{BO}}=w-\left( 1-\pi\right)v_{b}-\pi[\left( v_{b}-e_{p} \right)F\left( Q_{BO}-q_{p} \right)+\left( g-e_{c} \right)\left( 1-F\left( Q_{BO}+q_{c} \right) \right)+\left( e_{p}-e_{c} \right)F\left( Q_{BO} \right)+e_{c}]$ | (S1.1) |
| --- | --- |
| $\frac{\partial E({TC}_{R}(Q_{BO},q_{p},q_{c})}{\partial q_{p}}=o_{p}+\left( 1-\pi\right){(v}_{b}-e_{p})-\pi[\left( e_{p}{-v}_{b} \right)F\left( Q_{BO}-q_{p} \right)]$ | (S1.2) |
| $\frac{\partial E({TC}_{R}(Q_{BO},q_{p},q_{c})}{\partial q_{c}}=o_{c}+\pi[\left( e_{c}-g \right)\left( 1-F\left( Q_{BO}+q_{c} \right) \right)]$ | (S1.3) |

By setting the first derivative of the objective function equal to zero, the critical points of the HO's objective function are expressed as (S1.4) –(S1.6):

|  |  |  |
| --- | --- | --- |
| $Q_{BO}=F^{-1}(\frac{w+o_{p}-o_{c}-\pi e_{c}-\left( 1-\pi\right)e_{p}}{\pi\left( e_{p}-e_{c} \right)})$ | (S1.4) | |
| $q_{p}=Q-F^{-1}(\frac{o_{p}+\left( 1-\pi\right){(v_{b}-e}_{p})}{\pi\left( e_{p}-v_{b} \right)})$ | (S1.5) | |
| $q_{c}=F^{-1}\left( 1+\frac{o_{c}}{\pi\left( e_{c}-g \right)} \right)-Q$ | (S1.6) | |

To prove the convexity of the desired function, we form the Hessian matrix of the HO's objective function as follows:

| $\left[ \begin{matrix} \pi\{\left( e_{p}-v_{b} \right)f\left( Q_{BO}-q_{p} \right)+\left( g-e_{c} \right)f\left( Q_{BO}+q_{c} \right)+\left( e_{c}-e_{p} \right)f\left( Q_{BO} \right)\} & \pi\left( g-e_{c} \right)f\left( Q_{BO}+q_{c} \right) & \pi\{-\left( e_{p}-v_{b} \right)f\left( Q_{BO}-q_{p} \right) \\ \pi\left( g-e_{c} \right)f\left( Q_{BO}+q_{c} \right) & \pi\left( g-e_{c} \right)f\left( Q_{BO}+q_{c} \right) & 0 \\ \pi\{-\left( e_{p}-v_{b} \right)f\left( Q_{BO}-q_{p} \right) & 0 & \pi\{\left( e_{p}-v_{b} \right)f\left( Q_{BO}-q_{p} \right) \end{matrix} \right]$ | |
| --- | --- |
|  | (S1.7) |

Since$e_{c}>e_{p},e_{p}>v_{b},g>e_{c},f(Q_{BO}),f(Q_{BO}-q_{p}),f(Q_{BO}+q_{c})>0$ , the determinants of all first, second and third minors are positive and the HO's objective function is strictly convex. Hence, the critical points of the objective function are the optimal points of the HO's decisions.
