# Supplementary material for: Pricing and procurement strategies in the relief supply chain via bidirectional option contract
Source: PLoS One. 2026 Apr 1;21(4):e0341427. doi: 10.1371/journal.pone.0341427 (PMC13042840; doi:10.1371/journal.pone.0341427)
Supplement: S2 Appendix — (DOCX) [file pone.0341427.s002.docx]

**S2 Appendix. Proof of corollary 1**

Since$\frac{\partial Q_{BO}}{\partial o_{p}}=-\frac{1}{f\left( Q_{BO} \right) \pi\left( e_{c}-e_{p} \right)}$ and since ${(e}_{c})$ is always greater than$(e_{p}),f(Q_{BO})>0$, then $\frac{\partial Q_{BO}}{\partial o_{p}}<0$ and ${(Q}_{BO})$ has a negative relationship with${(o}_{p})$.

Additionally, since

$\frac{\partial Q_{BO}}{\partial o_{c}}=\frac{1}{f\left( Q_{BO} \right) \pi(e_{c}-e_{p})},\frac{\partial Q_{BO}}{\partial e_{c}}=\frac{(e_{c}+o_{c}-o_{p}-w)}{f\left( Q_{BO} \right) \pi(e_{c}-e_{p})^{2}},\frac{\partial Q_{BO}}{\partial e_{p}}=\frac{({o_{p}+w-e}_{p}-o_{c})}{f\left( Q_{BO} \right) \pi(e_{c}-e_{p})^{2}}$

And

$$e_{c}>e_{p},e_{p}>v_{b},g>e_{c},e_{c}+o_{c}>w+o_{p},e_{p}-o_{p}<o_{c}-w,f(Q_{BO})>0$$

Then $\frac{\partial Q_{BO}}{\partial o_{c}},\frac{\partial Q_{BO}}{\partial e_{c}},\frac{\partial Q_{BO}}{\partial e_{p}}>0$ and $Q_{BO}$ has positive relationships with${(e}_{p})$,${(e}_{c})$ and${(o}_{c})$.

Since $\frac{\partial q_{p}}{\partial o_{p}}=\frac{\partial Q_{BO}}{\partial o_{p}}-\frac{1}{f\left( Q_{BO}-q_{p} \right) \pi(e_{p}-v_{b})}$ and since $(e_{p})$ is always greater than$\left( v_{b} \right),f\left( Q_{BO}-q_{p} \right)>0$ and $\frac{\partial Q_{BO}}{\partial o_{p}}<0$, then$\frac{\partial q_{p}}{\partial o_{p}}<0$ and $(q_{p})$ has a negative relationship with${(o}_{p})$.

Additionally, since

$\frac{\partial q_{p}}{\partial o_{c}}= \frac{\partial Q_{BO}}{\partial o_{c}},\frac{\partial q_{p}}{\partial e_{c}}=\frac{\partial Q_{BO}}{\partial e_{c}},\frac{\partial q_{p}}{\partial e_{p}}=\frac{\partial Q_{BO}}{\partial e_{p}}+\frac{(o_{p})}{f\left( Q_{BO}-q_{p} \right) \pi(e_{p}-v_{b})^{2}}$

And

$$\frac{\partial Q_{BO}}{\partial o_{c}},\frac{\partial Q_{BO}}{\partial e_{c}},\frac{\partial Q_{BO}}{\partial e_{p}}>0 ,e_{p}>v_{b},f\left( Q_{BO}-q_{p} \right)>0$$

Then $\frac{\partial q_{p}}{\partial o_{c}},\frac{\partial q_{p}}{\partial e_{c}},\frac{\partial q_{p}}{\partial e_{p}}>0$ and $(q_{p})$ has positive relationships with${(e}_{p})$,${(e}_{c})$ and${(o}_{c})$.

Since $\frac{\partial q_{c}}{\partial o_{p}}=-\frac{\partial Q_{BO}}{\partial o_{p}}$ and $\frac{\partial Q_{BO}}{\partial o_{p}}<0$ , $(q_{c})$ has a positive relationship with${(o}_{p})$.

Additionally, since

$\frac{\partial q_{c}}{\partial o_{c}}=\frac{1}{f\left( Q_{BO}+q_{c} \right) \pi(e_{c}-g)}- \frac{\partial Q_{BO}}{\partial o_{c}},\frac{\partial q_{c}}{\partial e_{c}}=-\frac{o_{c}}{f\left( Q_{BO}+q_{c} \right) \pi(e_{c}-g)^{2}}-\frac{\partial Q_{BO}}{\partial e_{c}},\frac{\partial q_{c}}{\partial e_{p}}=-\frac{\partial Q_{BO}}{\partial e_{p}}$

And

$$\frac{\partial Q_{BO}}{\partial o_{c}},\frac{\partial Q_{BO}}{\partial e_{c}},\frac{\partial Q_{BO}}{\partial e_{p}}>0 ,e_{c}<g,f\left( Q_{BO}+q_{c} \right)>0$$

Then $\frac{\partial q_{c}}{\partial o_{c}},\frac{\partial q_{c}}{\partial e_{c}},\frac{\partial q_{c}}{\partial e_{p}}<0$ and $(q_{c})$ has negative relationships with${(e}_{p})$,${(e}_{c})$ and${(o}_{c})$.
