# Supplementary material for: Pricing and procurement strategies in the relief supply chain via bidirectional option contract
Source: PLoS One. 2026 Apr 1;21(4):e0341427. doi: 10.1371/journal.pone.0341427 (PMC13042840; doi:10.1371/journal.pone.0341427)
Supplement: S3 Appendix — (DOCX) [file pone.0341427.s003.docx]

**S3 Appendix. Proof of corollary 2**

Since$\frac{\partial Q_{BO}}{\partial g}=\frac{\partial Q_{BO}}{\partial v_{b}}=0$, the quantity of prepositioned items is determined independently of the shortage cost and HO's salvage value. Moreover, since$\frac{\partial Q_{BO}}{\partial w}=-\frac{1}{f\left( Q_{BO} \right)\pi(e_{c}-e_{p})}<0$, with an increase in the wholesale price, the HO prepositions fewer relief items. Given that $\frac{\partial Q_{BO}}{\partial\pi}=\frac{(o_{p}+w{-e}_{p}-o_{c})}{f\left( Q_{BO} \right)\pi^{2}(e_{c}-e_{p})}>0$, with an increase in the occurrence probability of a disaster, the HO prepositions more items in the pre-disaster phase to reduce the risk of supply shortage and avoid incurring higher post-disaster procurement costs. Since $\frac{\partial q_{p}}{\partial g}=\frac{\partial Q_{BO}}{\partial g}=0$, the value of $(q_{p})$ is independent of $(g)$. Since $\frac{\partial q_{p}}{\partial v_{b}}=\frac{\partial Q_{BO}}{\partial v_{b}}=-\frac{o_{p}}{f\left( Q_{BO}-q_{p} \right)\pi(e_{p}-v_{b})}<0$ with an increase in the HO's salvage value, the HO seeks to reduce $(q_{p})$ in order to lower the costs and risks. $\frac{\partial q_{p}}{\partial w}=\frac{\partial Q_{BO}}{\partial w}=-\frac{1}{f\left( Q_{BO} \right) \pi(e_{c}-e_{p})}<0$, therefore, with an increase in the wholesale price, the HO reserves fewer put options because it has prepositioned fewer items in its warehouses. Given that$\frac{\partial q_{p}}{\partial\pi}=\frac{\partial QQ_{BO}}{\partial\pi}-\frac{(e_{p}-o_{p}-v_{b})}{f\left( Q_{BO}-q_{p} \right) \pi^{2}(e_{p}-v_{b})}$ ,$\frac{\partial Q_{BO}}{\partial\pi}>0$ and$-\frac{(e_{p}-o_{p}-v_{b})}{f\left( Q_{BO}-q_{p} \right) \pi(e_{p}-v_{b})}<0$, it is not possible to comment on the relationship between the changes in ${(q}_{p})$ and$(\pi)$.

Since$\frac{\partial q_{c}}{\partial v_{b}}=-\frac{\partial Q_{BO}}{\partial v_{b}}=0$, the value of $(q_{c})$ is independent of$(v_{b})$. Since $\frac{\partial q_{c}}{\partial w}=-\frac{\partial Q_{BO}}{\partial w}=\frac{1}{f\left( Q_{BO} \right)\pi\left( e_{c}-e_{p} \right)}>0$ and $\frac{\partial q_{c}}{\partial g}=\frac{o_{c}}{f\left( Q_{BO}+q_{c} \right) \pi(e_{c}-g)^{2}}>0$, changes in $(q_{c})$ with respect to $(w)$ and $(g)$ are positive. This is logical because as the wholesale price increases and wholesale orders decrease, the supplier reserves more call options to reduce the risk of shortage and ensure that the demand is fully met. In addition, with an increase in shortage costs, the HO reserves more call options to reduce potential costs. Since$\frac{\partial q_{c}}{\partial\pi}=-\frac{bo_{c}}{{f\left( Q_{BO}+q_{c} \right) \pi}^{2}(e_{c}-g)}-\frac{\partial Q_{BO}}{\partial\pi}$ , $\frac{bo_{c}}{{f\left( Q_{BO}+q_{c} \right) \pi}^{2}({g-e}_{c})}>0$ and $-\frac{\partial Q_{BO}}{\partial\pi}<0$, it is not possible to comment on the relationship between the changes in ${(q}_{c})$ and $(\pi)$.
