# Supplementary material for: Pricing and procurement strategies in the relief supply chain via bidirectional option contract
Source: PLoS One. 2026 Apr 1;21(4):e0341427. doi: 10.1371/journal.pone.0341427 (PMC13042840; doi:10.1371/journal.pone.0341427)
Supplement: S4 Appendix — (DOCX) [file pone.0341427.s004.docx]

**S4 Appendix. Proof of Proposition 2**

The supplier is the leader of the Stackelberg game between the HO and itself. Considering a uniform probability distribution function for demand in the interval $\left[ 0,b \right]$, the probability density function of demand is$f(x)=\frac{1}{b}$. Relations (S4.1) to (S4.3) are then obtained by placing the probability density function into equations (3) to (5).

| $Q_{BO}=\frac{b(w+o_{p}-o_{c}-\pi e_{c}-\left( 1-\pi\right)e_{p})}{\pi(e_{p}-e_{c})}$ | (S4.1) |
| --- | --- |
| $q_{p}=Q-\frac{{b(o}_{p}+\left( 1-\pi\right)(v_{b}-e_{p}))}{\pi(e_{p}-v_{b})}$ | (S4.2) |
| $q_{c}=b(1+\frac{o_{c}}{\pi\left( e_{c}-g \right)})-Q$ | (S4.3) |

Therefore, by substituting the optimal values obtained for the HO in the supplier's objective function and differentiating with respect to ${(o}_{p})$ and$(o_{c})$, we have:

| (S4.4) | $\frac{\partial E\left( {TP}_{s}\left( o_{c},o_{p} \right) \right)}{\partial o_{c}}=b(-c\left( e_{c}-e_{p} \right)\left( e_{c}-g \right)-e_{p}g^{2}+2e_{p}go_{c}-g^{2}o_{c}$  $+g^{2}o_{p}+e_{p}gv_{s}-e_{p}o_{c}v_{s}+g^{2}w+{e_{c}}^{2}\left( -e_{p}+o_{p}+v_{s}+w \right)-e_{c}\left( -o_{c}v_{s}+e_{p}\left( -2g+o_{c}+v_{s} \right)+g\left( 2o_{p}+v_{s}+2w \right) \right))/\pi\left( e_{c}-e_{p} \right)\left( e_{c}-g \right)^{2}$ |
| --- | --- |
| (S4.5) | $\frac{\partial E({TP}_{s}\left( o_{c},o_{p} \right))}{\partial o_{p}}=b(e_{c}\left( {e_{p}}^{2}+2o_{p}v_{b}+{v_{b}}^{2}-e_{p}\left( o_{p}+2v_{b} \right)-o_{p}v_{s} \right)+{e_{p}}^{2}\left( o_{c}-w \right)+{v_{b}}^{2}\left( o_{c}-o_{p}-w \right)+e_{p}\left( -2o_{c}v_{b}+o_{p}v_{s}+2v_{b}w \right))/\pi\left( e_{c}-e_{p} \right)\left( e_{p}-v_{b} \right)^{2}$ |

Solving the equations above, we have:

| (S4.6) | $o_{c}=(e_{c}-g)(v_{b}^{2}\left( g-v_{s}+c-e_{c} \right)-e_{p}v_{s}\left( g+c \right)+v_{s}^{2}\left( e_{p}-e_{c} \right)+\left( ce_{c}+w\left( g-e_{c} \right) \right)\left( e_{p}-2v_{b}+v_{s} \right)+2v_{b}e_{c}v_{s})/(v_{b}^{2}\left( e_{c}-2g+v_{s} \right)+g^{2}\left( 2v_{b}-v_{s}-e_{p} \right)+2v_{s}\left( e_{p}g-e_{c}v_{b} \right)+v_{s}^{2}\left( e_{c}-e_{p} \right))$ |
| --- | --- |
| (S4.7) | $o_{p}=\left( e_{p}-v_{b} \right)^{2}(c\left( e_{c}-g \right)-g^{2}-w\left( e_{c}+v_{s} \right)+g\left( v_{s}+2w \right))/(v_{b}^{2}\left( e_{c}-2g+v_{s} \right)+g^{2}\left( 2v_{b}-v_{s}-e_{p} \right)+2v_{s}\left( e_{p}g-e_{c}v_{b} \right)+v_{s}^{2}\left( e_{c}-e_{p} \right))$ |

To prove the optimality of the obtained points, the Hessian matrix of the supplier's expected profit is formulated as follows:

| (S4.8) | $\left[ \begin{matrix} \frac{b(-\left( e_{p}-g \right)^{2}+\left( e_{c}-e_{p} \right)\left( v_{s}-e_{p} \right))}{\pi\left( e_{c}-e_{p} \right)\left( e_{c}-g \right)^{2}} & \frac{b}{\pi\left( e_{c}-e_{p} \right)} \\ \frac{b}{\pi\left( e_{c}-e_{p} \right)} & \frac{b(-\left( v_{b}-e_{c} \right)^{2}+\left( e_{c}-e_{p} \right)\left( e_{c}-v_{s} \right))}{\pi\left( e_{c}-e_{p} \right)\left( e_{p}-v_{b} \right)^{2}} \end{matrix} \right]$ |
| --- | --- |

Since$\left| (e_{p}-g)^{2} \right|>\left| (e_{c}-e_{p})(v_{s}-e_{p}) \right|$, the determinant of the first minor is always a negative value.

Since $\left| -\left( e_{p}-g \right)^{2}+\left( e_{c}-e_{p} \right)\left( v_{s}-e_{p} \right) \right|>\left| \left( e_{c}-g \right)^{2} \right|$

and

$$\left| -\left( v_{b}-e_{c} \right)^{2}+\left( e_{c}-v_{s} \right)\left( e_{c}-e_{p} \right) \right|>\left| \left( e_{p}-v_{b} \right)^{2} \right|$$

The determinant of the second minor is always greater than zero. Based on the values of the determinants of the Hessian function's minors, the supplier's objective function is strictly concave and the obtained critical points are the optimal points of the objective function.
