# Supplementary material for: Pricing and procurement strategies in the relief supply chain via bidirectional option contract
Source: PLoS One. 2026 Apr 1;21(4):e0341427. doi: 10.1371/journal.pone.0341427 (PMC13042840; doi:10.1371/journal.pone.0341427)
Supplement: S5 Appendix — (DOCX) [file pone.0341427.s005.docx]

**S5 Appendix.** **Proof of corollary 3**

If we substitute the relation $(v_{s}=v_{b})$ in the optimal values of ${(o}_{p})$ and${(o}_{c})$, we have:

| (S5.1) | $o_{c}=\frac{\lambda\left( g-w \right)\left( -gv_{s}+{v_{s}}^{2} \right)+\lambda gw-\lambda w^{2}+c(\left( 1-\lambda\right)g-v_{s}\lambda w))}{{(g-v_{s})}^{2}}$ |
| --- | --- |
| (S5.2) | $o_{p}=\frac{(-1+\lambda)\left( g-w \right)\left( -v_{s}+w \right)(-g-\lambda c+v_{s}+\lambda w)}{{(g-v_{s})}^{2}}$ |

By substituting the optimal reservation prices of call and put options and other parameters in the HO's optimal decisions, we have:

| (S5.3) | $Q_{BO}=\frac{b(-{v_{s}}^{2}+\lambda c\left( g-w \right)+v_{s}w+\lambda w^{2}+\pi\left( g^{2}+{v_{s}}^{2} \right)-g\left( -v_{s}+\left( 1+\lambda\right)w+2\pi v_{s} \right))}{\pi{(g-v_{s})}^{2}}$ |
| --- | --- |
| (S5.4) | $q_{p}=0$ |
| (S5.5) | $q_{c}=\frac{b(w-c)}{\pi(g-v_{s})}$ |
