# Supplementary material for: Pricing and procurement strategies in the relief supply chain via bidirectional option contract
Source: PLoS One. 2026 Apr 1;21(4):e0341427. doi: 10.1371/journal.pone.0341427 (PMC13042840; doi:10.1371/journal.pone.0341427)
Supplement: S6 Appendix — (DOCX) [file pone.0341427.s006.docx]

**S6 Appendix. Proof of Proposition 3**

By differentiating the HO's objective function with respect to $(Q_{w})$ and setting it equal to zero, we have:

| (S6.1) | $\frac{\partial(E({TC}_{R}\left( Q_{w} \right))}{\partial Q_{w}}=w-\left( 1-\pi\right)v_{b}-\pi v_{b}F\left( Q_{w} \right)+\pi gF\left( Q_{w} \right)-\pi g=0$ |
| --- | --- |

The order quantity is obtained by solving the equation above.

Since$\frac{\partial^{2}(E(TC_{R}(Q_{w})))}{\partial^{2}Q_{w}}= \pi(g-v_{b})\geq0$, the HO's objective function is strictly convex and the critical point obtained from the first derivative is the optimal point of the objective function.
