# Supplementary material for: Pricing and procurement strategies in the relief supply chain via bidirectional option contract
Source: PLoS One. 2026 Apr 1;21(4):e0341427. doi: 10.1371/journal.pone.0341427 (PMC13042840; doi:10.1371/journal.pone.0341427)
Supplement: S7 Appendix — (DOCX) [file pone.0341427.s007.docx]

**S7 Appendix. Proof of corollary 4**

Since $\frac{\partial\left( Q_{w} \right)}{\partial v_{b}}=\frac{g-w}{{f\left( Q_{w} \right)\left( g-v_{b} \right)}^{2}}\geq0$ and$\frac{\partial\left( Q_{w} \right)}{\partial g}=\frac{w-v_{b}}{f\left( Q_{w} \right)\left( g-v_{b} \right)^{2}}\geq0$, therefore ${(Q}_{w})$ has positive relationships with$(v_{b}$) and$(g$). On the other hand, since$\frac{\partial\left( Q_{w} \right)}{\partial w}=\frac{-1}{f\left( Q_{w} \right) \left( g-v \right)}\leq0$, $(Q_{w})$ has a negative relationship with$(w$).
