# Supplementary material for: Pricing and procurement strategies in the relief supply chain via bidirectional option contract
Source: PLoS One. 2026 Apr 1;21(4):e0341427. doi: 10.1371/journal.pone.0341427 (PMC13042840; doi:10.1371/journal.pone.0341427)
Supplement: S8 Appendix — (DOCX) [file pone.0341427.s008.docx]

**S8 Appendix. Proof of Proposition 4**

Due to the nonlinearity of the objective function in centralized mode, we obtain the first derivative of the objective function with respect to$(Q_{cs})$ as follows:

| (S8.1) | $\frac{\partial({TP}_{CS}(Q_{CS}))}{\partial Q_{Cs}}=-c+\left( 1-\pi\right)v_{s}+\pi\{v_{s}F\left( Q_{CS} \right)+g-gF\left( Q_{CS} \right)\}$ |
| --- | --- |

By setting the equation above equal to zero, the critical point of the function is obtained as follows:

| (S8.2) | $Q_{CS}=F^{-1}(\frac{b\left( c-\left( 1-\pi\right)v_{s}-\pi g \right)}{\pi\left( v_{s}-g \right)})$ |
| --- | --- |

As for global optimality, since the second derivative of the objective function with respect to $(Q_{cs})$ is$\pi(v_{s}-g)f(Q_{cs})<0$, the objective function is strictly concave, and the obtained critical point is the optimal point of the function.
