# Supplementary material for: Pricing and procurement strategies in the relief supply chain via bidirectional option contract
Source: PLoS One. 2026 Apr 1;21(4):e0341427. doi: 10.1371/journal.pone.0341427 (PMC13042840; doi:10.1371/journal.pone.0341427)
Supplement: S9 Appendix — (DOCX) [file pone.0341427.s009.docx]

**S9 Appendix.** **Proof of Corollary 5**

Since $\frac{\partial Q_{cs}}{\partial c}=\frac{1}{f\left( Q_{cs} \right)\pi\left( v_{s}-g \right)}<0$ , $\frac{\partial Q_{cs}}{\partial g}=\frac{\left( c-v_{s} \right)}{f\left( Q_{cs} \right)\pi\left( g-v_{s} \right)^{2}}>0$ , $\frac{\partial Q_{cs}}{\partial v_{s}}=\frac{\left( g-c \right)}{f(Q_{cs})\pi\left( g-v_{s} \right)^{2}}>0$ , and $\frac{\partial Q_{cs}}{\partial\pi}=\frac{\left( c-v_{s} \right)}{f\left( Q_{cs} \right)\pi^{2}\left( g-v_{s} \right)}>0$ , therefore ${(Q}_{cs})$ has an inverse relationship with the changes of $(c)$ and direct relationships with the changes of $(g)$, ${(v}_{s})$, and $(\pi)$.
